# The effectiveness of working memory training with individuals with intellectual disabilities – a meta-analytic review

**DOI:** 10.3389/fpsyg.2015.01230

**Published:** 2015-08-17

**Authors:** Henrik Danielsson, Valentina Zottarel, Lisa Palmqvist, Silvia Lanfranchi

**Affiliations:** ^1^Department of Behavioural Sciences and Learning, Linköping UniversityLinköping, Sweden; ^2^The Swedish Institute for Disability ResearchLinköping, Sweden; ^3^Department of Developmental Psychology and Socialisation, University of PadovaPadova, Italy

**Keywords:** intellectual disabilities, working memory training, visuo-spatial working memory, short-term memory, strategy training

## Abstract

Working memory (WM) training has been increasingly popular in the last years. Previous studies have shown that individuals with intellectual disabilities (ID) have low WM capacity and therefore would benefit by this type of intervention. The aim of this study was to investigate the effect of WM and cognitive training for individuals with ID. The effects reported in previous studies have varied and therefore a meta-analysis of articles in the major databases was conducted. Inclusion criteria included to have a pretest–posttest design with a training group and a control group and to have measures of WM or short-term memory. Ten studies with 28 comparisons were included. The results reveal a significant, but small, overall pretest–posttest effect size (ES) for WM training for individuals with ID compared to controls. A mixed WM approach, including both verbal and visuo-spatial components working mainly on strategies, was the only significant training type with a medium ES. The most commonly reported training type, visuo-spatial WM training, was performed in 60 percent of the included comparisons and had a non-significant ES close to zero. We conclude that even if there is an overall effect of WM training, a mixed WM approach appears to cause this effect. Given the few studies included and the different characteristics of the included studies, interpretations should be done with caution. However, different types of interventions appear to have different effects. Even if the results were promising, more studies are needed to better understand how to design an effective WM intervention for this group and to understand if, and how, these short-term effects remain over time and transfer to everyday activities.

## Introduction

Working memory (WM) has been defined as a system for the temporary holding and manipulation of information during the performance in a range of cognitive tasks (Baddeley, 1986). Until now, the critical role of WM in everyday life (e.g., reading, writing, arithmetic, learning, language-processing, orientation, imagination) and for individuals with intellectual disabilities (ID) has been shown in an impressive number of studies (for a review, see Baddeley, 1986).

One theoretical framework often used in research that assesses short-term memory (STM) and WM in individuals with ID, is Baddeley’s model (Baddeley and Hitch, 1974; Baddeley, 2000).

This model comprises of four components. The *central executive*, that can be seen as a limited-capacity processor responsible for attentional control over actions and for processing and coordinating the two slave systems called the *phonological loop* (for retaining linguistic information), and the *visuo-spatial sketchpad* (for retaining visuo-spatial information). Finally, the *episodic buffer*, added more recently to the model, is a multidimensional storage system that binds information from different sources in a unique code (Baddeley, 2000).

The distinction between the central executive system and specific memory storage systems (i.e., the phonological loop and the visuo-spatial sketchpad) is, in some ways, parallel to the distinctions between WM and STM.

A number of tasks involving both verbal and non-verbal material have been used so far to assess WM and STM. Experimental tasks assessing WM and the influence of the central executive component typically involve storage, processing, and effortful mental activity (Miyake and Shah, 1999; Kail and Hall, 2001). In contrast, STM tasks typically involve situations where participants passively retain small amounts of material, and minimal resources from long-term memory are activated to perform the task. STM tasks involve participants reproducing items in the order they were presented immediately after their presentation, and no cognitive processing is required (digit or word span forward tasks).

Several studies have previously shown the relationship between WM and intelligence, starting from the pioneering work of Just and Carpenter (1992). They found that intellectual performance may be enhanced if the individual is able to maintain more information in a temporary store and to simultaneously process it. Subsequently a series of correlational studies found high correlations between WM and intelligence (e.g., Kyllonen and Christal, 1990; Kane et al., 2005; Oberauer et al., 2005). In particular it has been shown that WM tasks, but not STM ones, are significantly related to intelligence, when the common variance reflecting the storage component present in both of them is removed (Engle et al., 1999).

Moreover, WM showed a predictive power for intellectual performance (Belacchi et al., 2010), as well as academic achievement areas such as literacy and numeracy (Alloway and Alloway, 2010), school readiness (Fitzpatrick and Pagani, 2012), and mathematical skills (Alloway and Passolunghi, 2011).

Previous studies have shown that individuals with ID have lower WM not only compared with typically developing individuals of the same chronological age (Henry, 2001; Henry and MacLean, 2002; Hasselhorn and Mähler, 2007; Van der Molen et al., 2007, 2009; Alloway, 2010; Schuchardt et al., 2010), but, at least in some aspects, even compared with typically developing children of the same mental age (Henry and MacLean, 2002; Van der Molen et al., 2007, 2009; Henry and Winfield, 2010).

Deficits were reported in verbal STM (Russell et al., 1996; Henry and MacLean, 2002; Lanfranchi et al., 2002; Bayliss et al., 2005; Van der Molen et al., 2007, 2009; Henry and Winfield, 2010; Schuchardt et al., 2010) and in WM (Lanfranchi et al., 2002; Danielsson et al., 2012), while visuo-spatial STM seems to be relatively preserved (Henry and MacLean, 2002; Rosenquist et al., 2003; Van der Molen et al., 2007, 2009; Henry and Winfield, 2010; Schuchardt et al., 2010).

However, contrasting findings regarding this tentative profile have been found (e.g., Bayliss et al., 2005; Hasselhorn and Mähler, 2007; Van der Molen et al., 2007), suggesting there probably is no unique profile for individuals with ID, but rather that other variables should also be considered. For example, Henry (2001) suggest that the level of severity might determine what areas are affected, with only verbal STM affected in individuals that have borderline ID and all STM and WM aspects impaired in individuals with mild ID.

Moreover, specific etiologies might have a particular STM/WM profile. For example, it has been shown that individuals with Down syndrome have an impaired verbal STM (e.g., Lanfranchi et al., 2004) in both verbal and visuo-spatial WM (e.g., Lanfranchi et al., 2012) while visuo-spatial STM seems to be relatively preserved, at least in his sequential component (e.g., Carretti et al., 2013). On the contrary, individuals with William’s syndrome showed a relatively preserved verbal STM and a relatively impaired visuo-spatial STM (e.g., Jarrold et al., 1999). Although, also in this case, both verbal and visuo-spatial WM were impaired (e.g., Lanfranchi et al., 2014). Finally, a profile of selective impairment only, in both verbal and visuo-spatial WM, has been found in individuals with Fragile X syndrome (Lanfranchi et al., 2009).

Taken together, these results suggest that at least some aspects of STM and/or WM are impaired even with respect to mental age in individuals with ID.

Considering the before-mentioned relationship established between WM and intelligence, academic achievement and everyday life, we believe that it is very important to verify whether it is possible to effectively train this important cognitive function in individuals with ID.

A previous meta-analytical study, addressed the more general question whether WM training is effective or not (Melby-Lervåg and Hulme, 2012). The results were not too optimistic, showing that the programs produced short-term improvement in WM, but these gains were not always maintained at the follow-up and were not generalized to other skills. One limit of the Melby-Lervåg and Hulme (2012) review is that it includes different types of clinical conditions. For this reason the aim of the present study is to perform a meta-analytic review only on individuals with ID in order to assess the effect of WM training, considering, the effect on the specific ability directly trained, so called direct effect, and effects on other types of WM and STM, so called near-transfer effects.

## Materials and Methods

### Protocol and Registration

This meta-analysis was conducted following the directions of “Practical meta-analysis” written by Lipsey and Wilson (2000) and Preferred Reporting Items for Systematic Reviews and Meta-Analyses statement by Moher et al. (2009).

### Eligibility Criteria

In the present paper we considered all the studies where at least one of the WM components, as described by Baddeley (2000) model, was trained. For this reasons, we considered training that works on verbal STM (phonological loop), visuo-spatial STM (visuo-spatial sketch pad) and verbal and visuo-spatial WM (central executive).

To be included, a study had to consider a STM/WM intervention (that could be verbal, visuo-spatial or mixed) and use a design that allowed training effects to be tested. This meant at least having a pretest–posttest design, and a training- and a control group. The study had to include measures of WM and/or STM. Participants were individuals below the age of 30, in order to avoid confounding due to the early cognitive decline that often occurs in this population. Participants should also have an IQ below 70 (according to one of the criteria for diagnosing ID) or declared as having ID or Borderline Intellectual Functioning. Individuals with Borderline Intellectual Functioning were also included, since a growing body of literature shows that the profile of memory deficits in this population is similar to that of individuals with ID (e.g., Alloway, 2010; Schuchardt et al., 2010).

Although we agree with the methodological issues in studies of WM training raised by Melby-Lervåg and Hulme (2012), we decided to include both randomized and non-randomized studies, as well as studies with treated and untreated control groups.

### Information Sources, Search Strategy, Literature Search

Electronic databases (Science Direct, Scopus, Pubmed, Web of Science, and Psycinfo) were searched. The following keyword for the electronic databases search were used: (cognitive enrichment OR cognitive improvement OR cognitive intervention OR cognitive training OR WM training) AND (developmental disorder OR ID OR intellectual disability OR intellectual disorder OR intellectual incapacities OR intellectual incapacity OR mental retardation OR Down syndrome OR Fragile × syndrome OR Prader Willi syndrome OR Williams syndrome) AND (child OR childhood OR children OR development OR developmental OR juvenile OR youth NOT adult). The search was conducted on September 11, 2014 and results were imported to the reference management system Mendeley^1^ where duplicates were removed. Literature was searched also by scanning reference lists, searching in prior reviews and personal requests to researcher in the field.

**FIGURE 1:**
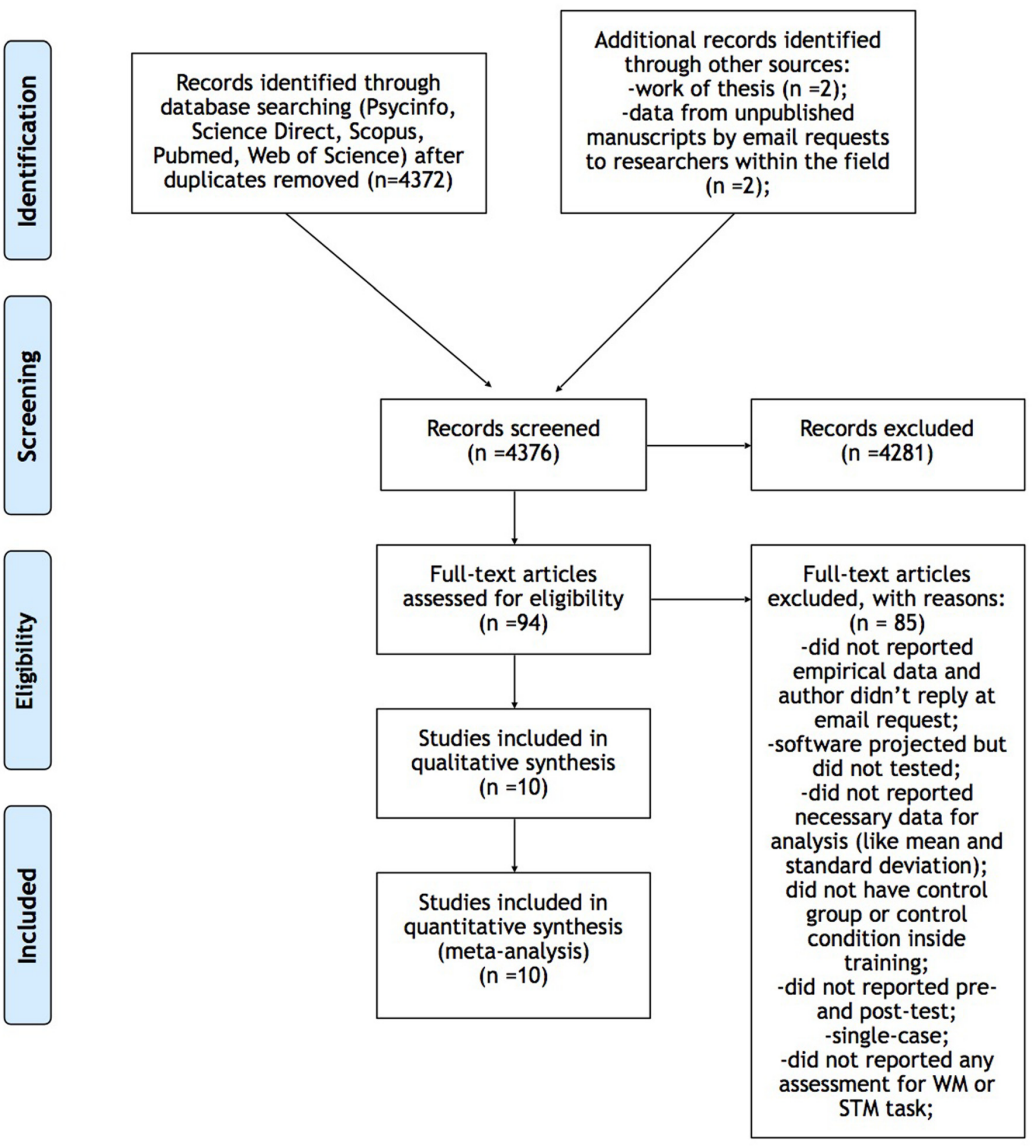
**Details about the literature search method and the criteria for inclusion and exclusion of studies**.

**Figure 1** shows details about the literature search method and the criteria for inclusion and exclusion of studies.

### Procedure

The focus on this meta-analysis is the direction and magnitude of the effects across studies, which is represented by the effect size (ES). The ES is, according to Lipsey and Wilson (2000), more suited for meta-analyses than significance testing. Effect sizes standardize findings across studies such that they can be directly compared, regardless of sample size or usage if study measures differs. This meta-analysis followed Lipsey and Wilson (2000) and used the *Standardized Mean Difference* (*d*) as ES for all included studies. The choice to use *d* was based on: (1) the included studies have group contrasts on the dependent variable, (2) all dependent variables are (i) inherently continuous and (ii) measured on a continuous scale, plus, (3) the included studies used different measures and scales. The ES was also corrected for a small sample upwardly bias (Lipsey and Wilson, 2000). Hedges (1981) concluded that the correction is necessary for all studies with *n* < 20. In order to use a consistent formula for all ESs in this meta-analysis, the correction is made even when the sample size is larger than *n* = 20. Rather than to use the pooled SD to calculate the ES (which is recommended by Lipsey and Wilson, 2000), the SD from the control group was used; it can be assumed that the variation is larger in the experimental group due to natural heterogeneity in the population. In cases where there was more than one ES per group, the mean ES is calculated as in Lipsey and Wilson (2000; s. 102).

A Random Effects Model was used to calculate the analog to the ANOVA analyses, which is preferable to use prior to the fixed model, since we can assume that the mean of the super population is different in training studies (Lipsey and Wilson, 2000).

## Results

Information about the included studies can be found in **Table 1**, which includes mean age, number of participants, participant diagnosis, type of training, and control treatment. As can be seen in **Table 1**, there were large differences between studies on all listed variables. The pretest–posttest ESs for all studies, both for the training group and the training group minus control group, can be found in **Table 2**.

**Table 1:** Characteristics of the working memory (WM) training studies included in the meta-analysis.

Study	Mean age training group	Mean age control group	*n* training group	*n* control group	Participants diagnosis	Type of training	Control treatment
Atia (2010)	30.1	30.1	7	6	Intellectual disabilities (ID)	VS WM	Untreated
Bennett et al. (2013)	9.5	9.5	10	11	Down syndrome	VS WM	Untreated
Conners et al. (2001)	10.8	10.8	6	5	Down syndrome	Verb WM	Visual activity
Danielsson et al. (2008)^1^	11.4	11.2	25	28	ID	VS WM	Math activity
Moalli (2006)	13.6	14.3	12	18	Down syndrome	Mixed WM	Knoledge on memory
Moalli et al. (2004)	13.8	12.6	8	8	Down syndrome	Mixed WM	Knowledge on memory
Pérez Sánchez et al. (2006)	21.5	22	10	10	Down syndrome	Verb short-term memory (STM)	Computer class
Smith and Jarrold (2014)^1^	16.2	14.0	9	8	Down syndrome	Verb STM	Visual activity
Söderqvist et al. (2012)	9.7	9.7	22	19	ID	VS WM	Non-adaptive memory training
Van der Molen et al. (2010)	15.2	15.3	41	27	Borderline intellectual functioning	VS WM	Non-adaptive memory training

*^1^This is a poster presented at a conference. Additional data has been provided by the authors after email request*.

**Table 2:** Pretest–posttest effect sizes (ESs) both for the training group and the training group minus control group analyses.

Study	Training type	Test type	Training group			Control group included		
			Cohen’s *d*	Lower C.I.	Upper C.I.	Cohen’s *d*	Lower C.I.	Upper C.I.
Atia (2010)	VS WM	VS WM	0.75	-0.33	1.83	-0.29	-1.43	0.86
Atia (2010)	VS WM	VS STM	0.55	-0.52	1.61	0.24	-0.87	1.34
Bennett et al. (2013)	VS WM	Verb WM	0.25	-0.63	1.13	0.55	-0.30	1.40
Bennett et al. (2013)	VS WM	VS WM	0.98	0.05	1.91	0.95	0.07	1.82
Bennett et al. (2013)	VS WM	Verb STM	0.12	-0.75	1.00	0.17	-0.67	1.02
Bennett et al. (2013)	VS WM	VS STM	0.61	-0.28	1.51	0.87	0.01	1.73
Conners et al. (2001)	Verb WM	Verb WM	0.61	-0.56	1.77	0.04	-1.18	1.26
Conners et al. (2001)	Verb WM	VS WM	0.28	-0.85	1.42	0.54	-0.65	1.73
Danielsson et al. (2008)	VS WM	Verb WM	-0.05	-0.79	0.69	-0.27	-1.02	0.49
Danielsson et al. (2008)	VS WM	VS WM	-0.84	-1.61	-0.07	-0.06	-0.84	0.73
Danielsson et al. (2008)	VS WM	Verb STM	0.00	-0.74	0.74	-0.30	-1.05	0.46
Danielsson et al. (2008)	VS WM	VS STM	1.61	0.76	2.47	-0.28	-1.17	0.60
Moalli (2006)	Mixed WM	Verb WM	0.64	-0.09	1.37	0.03	-0.67	0.73
Moalli (2006)	Mixed WM	VS WM	0.99	0.23	1.76	0.99	0.28	1.70
Moalli (2006)	Mixed WM	Verb STM	0.51	-0.22	1.24	0.31	-0.38	1.01
Moalli (2006)	Mixed WM	VS STM	1.01	0.24	1.77	1.08	0.37	1.79
Moalli et al. (2004)	Mixed WM	Verb STM	0.51	-0.22	1.24	0.31	-0.38	1.01
Moalli et al. (2004)	Mixed WM	VS STM	1.01	0.24	1.77	1.08	0.37	1.79
Pérez Sánchez et al. (2006)	Verb STM	Verb STM	0.72	-0.19	1.63	0.74	-0.13	1.60
Pérez Sánchez et al. (2006)	Verb STM	VS STM	0.32	-0.56	1.21	0.30	-0.58	1.18
Smith and Jarrold (2014)	Verb STM	Verb STM	0.27	-0.66	1.20	0.03	-0.92	0.99
Söderqvist et al. (2012)	VS WM	Verb WM	0.30	-0.29	0.90	0.42	-0.19	1.04
Söderqvist et al. (2012)	VS WM	VS WM	0.42	-0.17	1.02	0.41	-0.21	1.03
Söderqvist et al. (2012)	VS WM	Verb STM	-0.30	-0.90	0.29	-0.67	-1.29	-0.06
Van der Molen et al. (2010)	VS WM	Verb WM	0.31	-0.18	0.80	0.12	-0.39	0.64
Van der Molen et al. (2010)	VS WM	VS WM	0.36	-0.13	0.85	-0.14	-0.66	0.38
Van der Molen et al. (2010)	VS WM	Verb STM	0.27	-0.21	0.76	0.09	-0.43	0.60
Van der Molen et al. (2010)	VS WM	VS STM	0.29	-0.20	0.78	-0.19	-0.71	0.33

The overall ES for the training group was 0.42, 95% CI (0.24,0.59), *p* < 0.001. When subtracting the ES from the control group (i.e., the placebo effect), the remaining effect was.24, 95% CI (0.06,0.43), *p* < 0.01. These ESs correspond to a medium and a small ES (Cohen, 1962) respectively. The ESs have been broken down on the two main variables, type of training and type of memory test. **Table 3** shows the results of these analyses. For the training group, there was a significant effect of visuo-spatial WM training [0.29, 95% CI (0.07,0.26), *p* < 0.001], which was driven by the significant effect on visuo-spatial STM [0.69, 95% CI (0.22,0.67), *p* < 0.05], whereas the effects on the other tests were non-significant. However, both these effects were non-significant and close to zero when subtracting the control groups ES.

**Table 3:** The ESs broken down on the two main variables, type of training and type of memory test.

	Training type			
Memory test	Visuo-spatial WM	Mixed WM	Verbal WM	Verbal STM
**Training group**				
Verbal WM	0.22 (0.22)	0.64 (0.58)	0.24 (0.60)^∧^	-
Visuo-spatial WM	0.29 (0.21)	0.99 (0.60)	0.28 (0.58)^∧^	-
Verbal STM	0.03 (0.22)	1.01 (0.46)^∗^	-	0.50 (0.33)
Visuo-spatial STM	0.69 (0.24)^∗^	0.84 (0.45)	-	0.33 (0.45)^∧^
Total training group	0.29 (0.11)^∗∗^	0.88 (0.26)^∗∗^	0.44 (0.42)	0.44 (0.27)
**Training group minus control group**				
Verbal WM	0.20 (0.20)	0.03 (0.60)	0.04 (0.62)	-
Visuo-spatial WM	0.17 (0.19)	0.99 (0.61)	0.54 (0.61)^∧^	-
Verbal STM	-0.18 (0.20)	0.83 (0.47)	-	0.42 (0.35)
Visuo-spatial STM	0.08 (0.23)	0.91 (0.46)	-	0.30 (0.45)
Total Training group minus control group	0.07 (0.10)	0.74 (0.15)^∗∗^	0.30 (0.43)	0.38 (0.26)

*Effect sizes at the top for the training group analysis and at the bottom for the training group minus control group analysis. SE is presented within brackets*.

*^∗^*p* < 0.05, ^∗∗^*p* < 0.001*.

*^∧^Only one ES; therefore not computed*.

In the training group, there was a significant overall effect of mixed WM training [0.88, 95% CI (0.37,0.99), *p* < 0.001], which was driven by a significant effect on verbal STM [1.01, 95% CI (0.11,0.67), *p* < 0.05]. The effects on the other test types were large, but not significant. In the training group minus control group analysis, the overall effect was still significant [0.74, 95% CI (0.45, 1.02), *p* < 0.001]. The effects on all test types were non-significant, but for all test types, except verbal WM, the ES was large.

**FIGURE 2:**
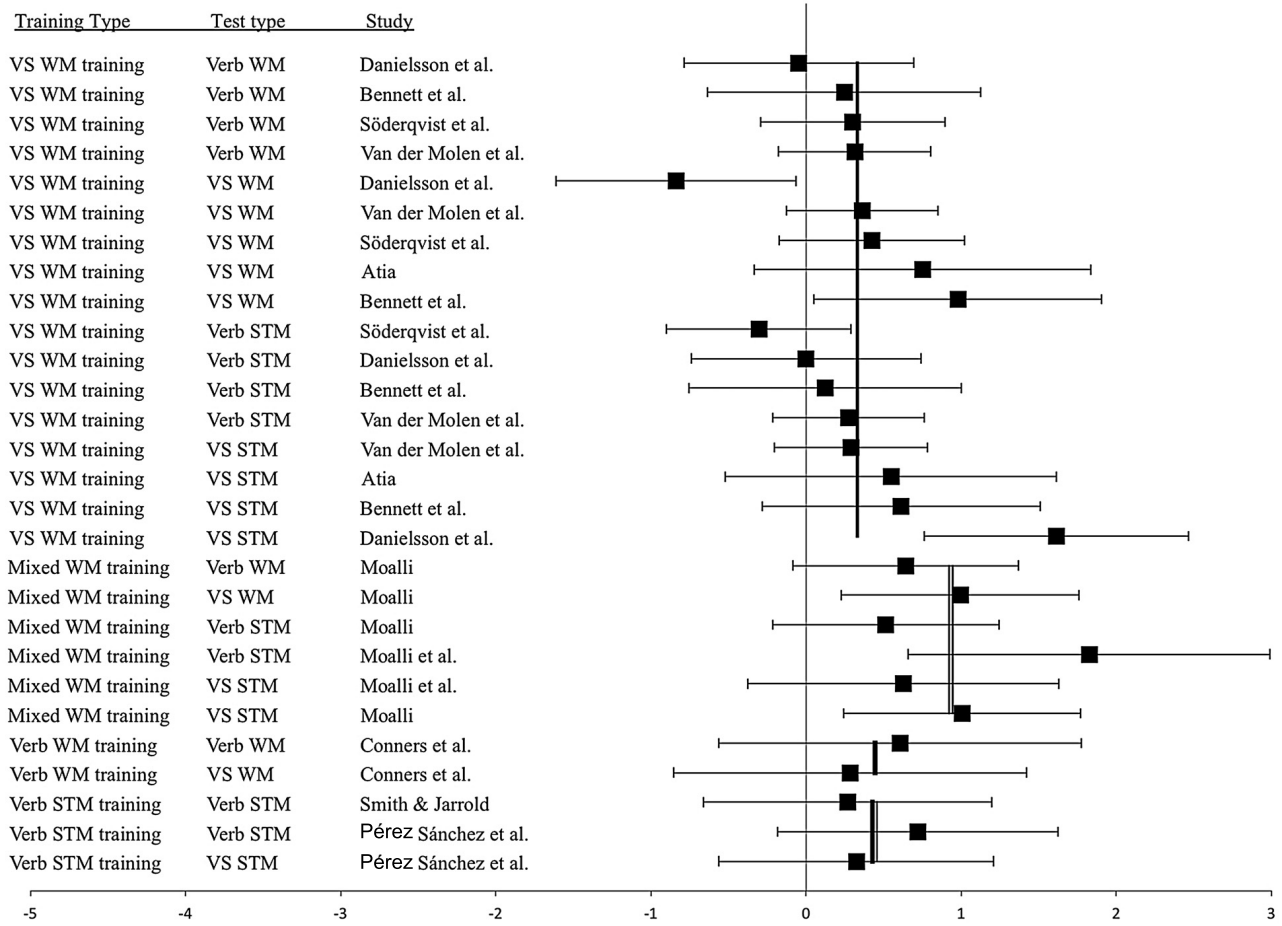
**Forrest plot for training group post-test minus pre-test effect sizes (ESs) sorted by type of training and test type**. The overall ES for each training type is displayed by a line. Type of training and type of test are listed in the two left columns.

**FIGURE 3:**
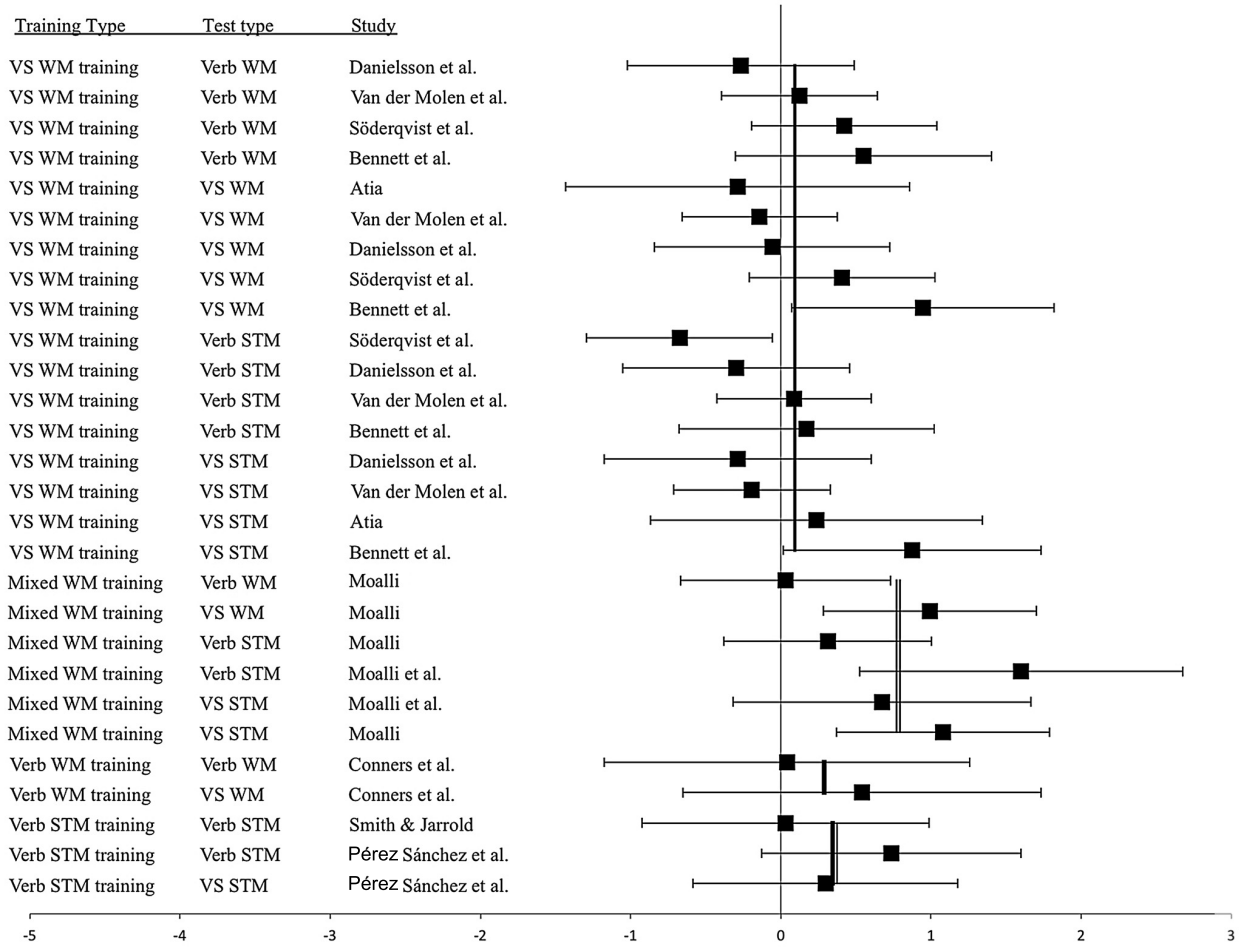
**Forrest plot for training group minus control group ESs sorted by type of training and test type**. The overall ES for each training type is displayed by a line. Type of training and type of test are listed in the two left columns.

The ESs for all studies are shown in **Figure 2** for the training group and in **Figure 3** for the training group minus control group. The studies are sorted by type of training and then by the magnitude of the ES. As can be seen, there were large variations in ESs and large confidence intervals in many cases. There were even studies where the confidence interval does not cover the overall ES for that type of training. This indicates that the included articles indeed have different characteristics, or that some studies could have low quality.

## Discussion

The results show overall effects on WM training for individuals with ID. This was true for pretest–posttest ESs for both the training group (medium ES) and the training group minus control group analyses (small ES). Several different types of WM training have been used but only mixed WM training, with both verbal and visuo-spatial components, showed significant training effects. A breakdown of the training effects on verbal and visuo-spatial WM and STM tests indicated somewhat larger ESs for the STM tests compared to the WM tests.

Taken together these results suggest that different types of WM training can lead to different outputs on STM and WM in individuals with ID, and that depending on the type of activities the training can be more or less effective.

From the data analyzed in this study a mixed memory training seems to be the more effective in improving WM in individuals with ID, leading to greater improvements on verbal and visuo-spatial STM, than on WM. Only two studies (Moalli et al., 2004 and Moalli, 2006) used a mixed training program and the training activities used in both studies were similar. The training was focused on helping the child learn different strategies to improve verbal STM and WM, and to understand when and how to use them in verbal and visuo-spatial STM and WM tasks. The training focused on a variety of STM and WM tasks in order to exercise the use of the newly learned strategies. From a theoretical point of view the results of this meta-analysis suggest that, if we consider individuals with ID as one group, a mixed training approach works better than training focusing only on one particular WM aspect. This could be due to that individuals with ID show deficits in both verbal and visuo-spatial STM/WM (e.g., Lanfranchi et al., 2002; Danielsson et al., 2012). Moreover, one hypothesis is that working in a “metacognitive way” helps the person to acquire new strategies and to learn when and how to use them, which produces better results than just exercising STM/WM.

From a statistical point of view it is more probable that interventions that target multiple components of WM are more effective given the individual differences in strengths and weaknesses for different components of WM. This is in line with a meta-analysis on WM training for children and adolescents with ADHD (Cortese et al., 2014) where interventions targeting multiple neuropsychological deficits had large effects on ADHD symptoms.

That the effect for visuo-spatial WM training was close to zero in the training minus control group analysis makes the interpretation problematic, since this type of training accounts for 60% of the included comparisons.

However, at least half of the studies (Moalli et al., 2004; Moalli, 2006; Van der Molen et al., 2010; Söderqvist et al., 2012; Smith and Jarrold, 2014) used a control group where the given memory training was supposed to be less effective than the target training. In Van der Molen et al. (2010) and Söderqvist et al. (2012) the control group was given a non-adaptive version of the target training. In Moalli et al. (2004) and Moalli (2006), the control group worked on the knowledge of how memory functions, and in Smith and Jarrold (2014) the control group worked on a visual activity that also involves memory. Although we agree with Melby-Lervåg and Hulme (2012), that an untreated control group might overestimate the effect due to the training, a control group that engage in activities that, in some way, involve memory, could have reduced the ES of the difference between the training and control group.

This meta-analysis highlights the lack of studies on WM training in individuals with ID. Although a number of studies have highlighted STM/WM deficit in individuals with ID (e.g., Lanfranchi et al., 2002; Danielsson et al., 2012), only few studies have explored the possibility to improve this important cognitive aspect in this population. Moreover, some of these few studies had to be excluded due to the methodological problems highlighted by Melby-Lervåg and Hulme (2012), such as the lack of a control group or lack of a pretest–posttest design. Therefore, we believe that future research should better explore the possibility to train WM in a population with ID, with a pretest–posttest design and an adequate control group.

## Limitations of the Current Study

This meta-analysis has several limitations. Since there were few studies in this area, there were several important differences between the studies. If there had been more studies, these could have been analyzed as moderator variables (for example, one study allowed for an IQ up to 85, which is outside the traditional definition of intellectual disability). There are groups with different causes to their ID, for example individuals with Down syndrome as well as individuals with intellectual disability for unknown cause. Even though the control group in each study always had the same participant characteristics as the training group, the control groups differed between studies since the training groups had different causes to their ID. The controls also differed in terms of what they did between pre- and posttest. Some were active controls, who did other types of training with different levels of similarity to the training of the training group and some were passive controls. In an effort to acknowledge the control group issues in this meta-analysis the results are reported both with and without subtraction of the control group. The pattern of results are relatively similar for both analyses, which indicates that the control groups have small, or at least relatively equally distributed, effects in the different analyses.

The meta-analysis was also limited to only close transfer effects, i.e., on WM and STM, since most studies did not include tests of far transfer and those who did had very different types of tests.

## Conclusion

This study shows that there was an overall significant effect of WM training for individuals with ID. An analysis of different types of training showed that only a mixed WM training approach, with both verbal and visuo-spatial components, had a significant ES. The effects were largest on STM tests. Even if the results are promising, they should be interpreted with caution since there were few studies included in the meta-analysis, the studies were relatively different with regard to type of intellectual disability, type of control groups and type of control group training.

The training effects analyzed are limited to effects to WM and STM test. The transfer to everyday activities and clinically relevant tasks have not been analyzed here due to very few of those measures in the studies. Meta-analysis on WM training for children with ADHD typically show an effect on WM, but limited transfer to clinically relevant tasks (e.g., Melby-Lervåg and Hulme, 2012; Rapport et al., 2013; Cortese et al., 2014). However, one meta-analysis (Spencer-Smith and Klingberg, 2015) actually found transfer to one activity, inattention in daily life. These results indicate that even if there are short-term effects on WM and STM for individuals with ID, these effects do not necessarily generalize to long-term effects or everyday life activities. More studies are needed to better understand how to design an effective WM intervention for this group and to understand if, and how these effects transfer to everyday activities.

## Conflict of Interest Statement

The authors declare that the research was conducted in the absence of any commercial or financial relationships that could be construed as a potential conflict of interest.

## Footnotes

1 www.mendeley.com
